# Modelling Favourability for Invasive Species Encroachment to Identify Areas of Native Species Vulnerability

**DOI:** 10.1155/2014/519710

**Published:** 2014-01-21

**Authors:** David Romero, José C. Báez, Francisco Ferri-Yáñez, Jesús J. Bellido, Raimundo Real

**Affiliations:** ^1^Departamento de Biología Animal, Facultad de Ciencias, Universidad de Málaga, 29071 Málaga, Spain; ^2^Instituto Español de Oceanografía, IEO, 29016 Málaga, Spain; ^3^Departamento de Biogeografía y Cambio Global, Museo Nacional de Ciencias Naturales, CSIC & Laboratorio Internacional en Cambio Global CSIC-PUC (LINCGlobal), Calle José Gutierrez Abascal 2, 28006 Madrid, Spain; ^4^Aula del Mar de Málaga, 29003 Málaga, Spain

## Abstract

We assessed the vulnerability of the native Mediterranean pond turtle to encroachment by the invasive red-eared slider in southern Spain. We first obtained an ecogeographical favourability model for the Mediterranean pond turtle. We then modelled the presence/absence of the red-eared slider in the Mediterranean pond turtle range and obtained an encroachment favourability model. We also obtained a favourability model for the red-eared slider using the ecogeographical favourability for the Mediterranean pond turtle as a predictor. When favourability for the Mediterranean pond turtle was high, favourability for the red-eared slider was low, suggesting that in these areas the Mediterranean pond turtle may resist encroachment by the red-eared slider. We also calculated favourability overlap between the two species, which is their simultaneous favourability. Grids with low overlap had higher favourability values for the Mediterranean pond turtle and, consequently, were of lesser conservation concern. A few grids had high values for both species, being potentially suitable for coexistence. Grids with intermediate overlap had similar intermediate favourability values for both species and were therefore areas where the Mediterranean pond turtle was more vulnerable to encroachment by the red-eared slider. We mapped the favourability overlap to provide a map of vulnerability of the Mediterranean pond turtle to encroachment by the red-eared slider.

## 1. Introduction

The effect of alien invasive species on native ecosystems is of high conservation concern because of the possible displacement of native species and the subsequent homogenization of global biodiversity [1]. Many authors have suggested that interspecific competition is one of the most important processes that determine the final outcome of biological invasions [2]. Therefore, the assessment of interactions between native and invasive species is crucial for predicting and preventing biological invasions [2, 3]. Thus, the analysis of the settlement of one exotic species in the distribution area of another similar native species could be helpful for understanding the synecological processes underlying biological invasions.

The Mediterranean pond turtle (*Mauremys leprosa*, Schweiger, 1812; Geoemydidae) is the most abundant Chelonia (Testudines) in the Iberian Peninsula. However, it is an endangered species whose populations have considerably declined during the last decades [4]. The distribution range includes the Iberian Peninsula and northwest Africa [5], and it can be found in different anthropogenic microhabitats and freshwater ecosystems [6, 7] such as rice fields or irrigation ditches [8]. The largest population of the species occurs in the Iberian Peninsula where it is classified as “vulnerable” in the Spanish red book [8].

The red-eared slider (*Trachemys scripta*, Schoepff, 1792; Emydidae) is an invasive species that is currently breeding in the Iberian Peninsula [9, 10]. Overall, the red-eared slider and the Mediterranean pond turtle occupy similar microhabitats and according to different studies the former is competing with and displacing the latter [11, 12]. Nevertheless, the nature of the competitive interactions between the native and invasive species is unclear [12, 13].

In this context, analysing the biogeographical interactions between the red-eared slider and the native Mediterranean pond turtle would increase our understanding of the distributional relationships between a native and a similar foreign species. The use of species distribution modelling is an appropriate approach for this objective, because if the competitive interactions between the introduced and the native species show any environmental trends, these should be reflected in ecogeographical models [14].

Our general aim is to provide a modelling methodology to identify areas of high conservation concern regarding a native species due to the encroachment of an invasive alien species. As a case study, we modelled the favourability for encroachment of the invasive red-eared slider into the area occupied by the native Mediterranean pond turtle to identify areas of vulnerability of the latter species. We proceeded in three steps: (1) we generated an encroachment favourability model for the red-eared slider within the range of the Mediterranean pond turtle to determine its potential to invade the area of the Mediterranean pond turtle; (2) we generated an ecogeographical favourability model for the native Mediterranean pond turtle; (3) we overlapped these two favourability models and calculated the mean favourability for each species in each overlap interval; and (4) we used these values to map the areas of conflict between the two species and the zones of more conservation concern regarding the Mediterranean pond turtle.

## 2. Methods

### 2.1. Study Area

**Figure 1:**
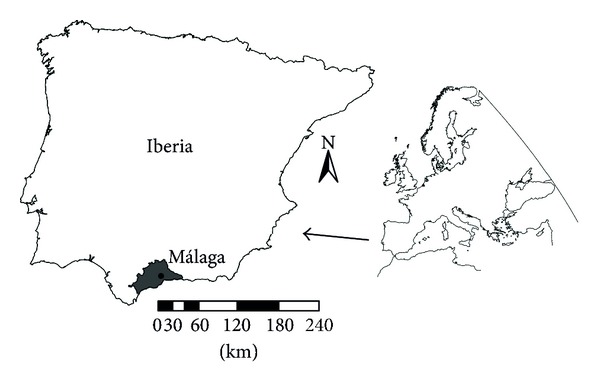
Study area represented in the European context. On the map on the left, grey shadows correspond to Málaga, within Iberia.

This paper focuses on Málaga province, in the south of the Iberian Peninsula (Figure 1). The native Mediterranean pond turtle is widely distributed in the area occupying different types of freshwater ecosystems [4, 15]. Málaga covers an area of about 7,300 km^2^ which puts it on the limit between the landscape and regional scales at which climate, topography, and land use are thought to control species distributions [16]. Málaga province has a population of more than 1,600,000 people [17]. The climate is affected by steep gradients due to the mountainous topography, with altitudes ranging from sea level to 2,000 meters a.s.l. The general climate is Mediterranean with hot dry summers and short mild winters. A subtropical Mediterranean climate prevails in the eastern coastal areas, whereas an oceanic Mediterranean climate is more evident in the west. Annual average temperatures range between 12.5°C and 19°C.

### 2.2. Species Distribution

**Figure 2:**
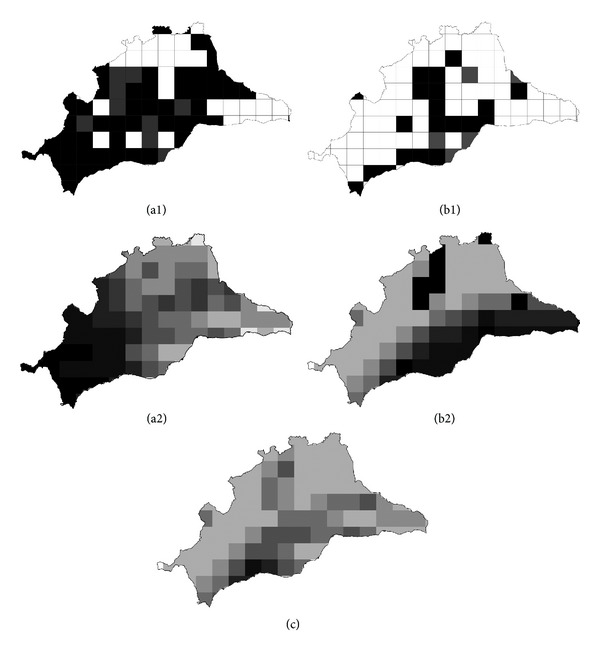
Maps (a1) and (b1) show the distribution of both species in Málaga. Filled cells show the presence of *Mauremys leprosa* in (a1) and of *Trachemys scripta* in (b1). Black cells correspond to presences taken from Pleguezuelos et al. [15], and gray cells represent presences found during a more recent survey [7]. Maps (a2) and (b2) show the *F*-*Mauremys*-Ecogeographical model and *F*-*Trachemys*-Encroachment model, respectively. Map (c) shows the intersection between the favourability of both species. Shading ranges from white to black, where white indicates completely unfavourable areas (0) and black indicates completely favourable areas (1).

Distribution data for both species (Figure 2, maps a1 and b1) came from an intensive survey in the study area (records published in Romero et al. [6, 7] and from Pleguezuelos et al. [15]). The two species studied occupied both lotic and lentic environments. The Mediterranean pond turtle occupied 72% of the study area, whereas the red-eared slider was found in 26% of the study area.

### 2.3. Variables

We used an initial pool of ecogeographic variables to identify the macroenvironmental factors that affect the turtles' distribution. We divided the variables into five explanatory factors: spatial situation, human activity, topography, climate, and land use (Table 1). We preferred to use a large biogeographical resolution scale rather than a local scale, as our aim was to describe the macroenvironmental processes around the sampling points that drive the distribution of both species rather than the ecological processes of each water reservoir that govern local occupancy. For this reason, we used a resolution of 100 km^2^ (104 cells in the study area). In this context, we expected that the distribution models based in ecogeographical factors will affect the potential distribution of both studied pond turtles. Specifically, we worked with 70 presence records of the Mediterranean pond turtle and 24 presence records of red-eared slider, 19 of which were taken from the Atlas and five were new records.

The procedure for obtaining the variables related to spatial distribution, topography, climate, and human activity has been described in Barbosa et al. [18], Castro et al. [19], and Muñoz et al. [20]. Land-use variables were extracted from Corine Land Cover [21]. We reclassified the categories into those shown in Table 1. The new land-cover classes, initially in polygon shape-file format, were processed using ESRI ArcMap 9.2 software. Polygons were converted to raster of 1000 × 1000 m using a spatial-analyst tool. Finally, the surface area of each category was calculated for each 100-km^2^ square in Málaga province.

### 2.4. The Favourability Models

We analysed the environmental factors that influence the cooccurrence of the two species, first modelling each species separately. We used univariate logistic regression to obtain the statistical potential occurrence of each species as a function of each ecogeographic variable [22–24] in order to determine the predictor variables that were significantly related to the distribution of each species. To control the increase in type I errors due to multiple tests [25], we only accepted those variables that were significant under a false discovery rate of *q* < 0.05, using the Benjamini and Hochberg procedure [25]. We then performed forward-backward stepwise logistic regression on the subset of significant predictor variables to obtain a multivariate logistic model. These models were named the *Mauremys*-Ecogeographical model and the *Trachemys*-Ecogeographical model.

We then performed a similar procedure to assess the presence or absence of the red-eared slider in the range of the Mediterranean pond turtle, with the aim of determining the factors that drive the encroachment of the red-eared slider into the range of the Mediterranean pond turtle. This model was named the *Trachemys*-Encroachment model.

We used the favourability function (*F*) to identify the areas that are favourable to the species [23], regardless of the presence/absence ratio. Favourability was easily calculated from the probability obtained from the logistic regressions according to the expression

(1) $$\begin{array}{c} F=\frac{\left[P/\left(1-P\right)\right]}{\left[\left(n1/n0\right)+\left(P/\left[1-P\right]\right)\right]}, \end{array}$$

where *P* is the probability of a species being present, *n*1 is the number of observed presences in the geographical region, and *n*0 is the number of observed absences. We applied this favourability function to the three models described above.

In addition, we obtained a synecological favourability model for the red-eared slider by performing a logistic regression of its presence/absence using the values of *F*-*Mauremys*-Ecogeographical model as a predictor variable and applying the favourability function to obtain the *F*-*Trachemys*-Synecological model.

### 2.5. Model Assessment

The goodness-of-fit of the models was assessed using the Hosmer and Lemeshow test (the test statistic also follows a chi-square distribution; low *P* values would indicate lack of fit of the model [26]). The discrimination capacity of the models was evaluated with the area under the curve (AUC) of the receiving operating Characteristic (ROC). To evaluate the classification capacity of the models, we obtained a set of measures of classification based on the 0.5-favourability threshold (as favourability is independent of prevalence, 0.5 is the favourability value at which both sensitivity and specificity are equal): the correct classification rate (CCR), sensitivity, specificity, and Cohen's Kappa [27].

### 2.6. Interspecific Relationships

We used Pearson's chi-squared test to determine whether the distribution of the two species overlapped more than would be expected at random. Based on the values of the *F*-*Mauremys*-Ecogeographical model and the *F*-*Trachemys*-Encroachment model, we calculated favourability overlap between the two species [14] in the *Mauremys* range, indicating the degree to which the local favourability is similar for the two species. To obtain this, we calculated the fuzzy intersection between the fuzzy sets of areas favourable to each species (minimum favourability value for the two species at a given location), which can be used to identify the fuzzy set of areas simultaneously favourable for the two species; we also calculated the fuzzy union (maximum favourability value), which can identify the fuzzy set of areas favourable to either species [28]. We obtained the favourability overlap as the intersection divided by the union [29, 30]. This overlap ranges from 0, indicating no overlap, to 1, indicating the highest favourability overlap. We calculated the mean favourability values in each overlap interval for each species. Finally, we used these values to map the areas of conflict between the two species and the zones of more conservation concern regarding the Mediterranean pond turtle.

## 3. Results 

We obtained a significant favourability model of the distribution of the Mediterranean pond turtle in Málaga province using all the available data, the *F*-*Mauremys*-Ecogeographical model, whose logit function was

(2) $$\begin{array}{c} Y=+2.572*\text{HumI}-1.871*\text{Lo}+0.040*\text{SE}-13.495. \end{array}$$

The variables that explained the distribution of the Mediterranean pond turtle were humidity index (HumI), longitude (Lo), and southward exposure degree (SE).

The model's goodness-of-fit statistic showed no significant differences between the observed and expected values (Hosmer and Lemeshow: chi-square = 5.513, df = 8, and *P* = 0.702).

Regarding the *F*-*Trachemys*-Ecogeographical model, no variable was significant under a false discovery rate of *q* < 0.05. For this reason, we could not obtain a model that performed significantly better than a randomly generated model. The presences of the red-eared slider tended to overlap with the *M. leprosa* presences, and the distributions of the two species had an overlap value higher than that expected at random (Pearson *χ* ^2^ = 9.3962, *P* value = 0.002174). We found a significant favourability model when analysing the distribution of the red-eared slider over the Mediterranean pond turtle range, the *F*-*Trachemys*-Encroachment model (Hosmer and Lemeshow: chi-square = 8.961, df = 8, and *P* = 0.346), with the following logit function:

(3) $$\begin{array}{c} Y=-0.370*\text{HJan}+1.881*\text{LK}+29.943. \end{array}$$

Thus, the variables that explained the distribution of the red-eared slider were mean relative air humidity in January (HJan) and reservoirs and lakes (LK).

Favourability maps resulting from both models are shown in Figure 2. According to the models, the most favourable conditions for the Mediterranean pond turtle in Málaga were located in the southwest of the province, whereas the most favourable conditions for the red-eared slider were in central to eastern Málaga.

The logit functions obtained for the *F*-*Trachemys*-Synecology model presented the form

(4) Y=+13.416∗F-Mauremys-Ecogeographical −11.452∗F-Mauremys-Ecogeographical2−4.431.

Discrimination and classification assessment of the three models are shown in Table 2. Regarding sensitivity, specificity, and CCR, the *F*-*Mauremys*-Ecogeographical model and the *F*-*Trachemys*-Encroachment model correctly classified the presences in grids of high favourability and correctly classified the absences in grids with low favourability. The *Mauremys* model was the best model for classifying presences and absences (highest Kappa, sensitivity, specificity, and CCR) and discrimination capacity (AUC) was always higher than 0.8, that is, excellent, according to Hosmer and Lemeshow [26]. The *F*-*Trachemys*-Synecological model had the highest sensitivity values and the lowest kappa, specificity, CCR, and AUC values.

We obtained the intersection (or minimum according to fuzzy logic [30]) between the Mediterranean pond turtle and the red-eared slider models; the areas of greatest interaction are shown in Figure 2(c).

**Figure 3:**
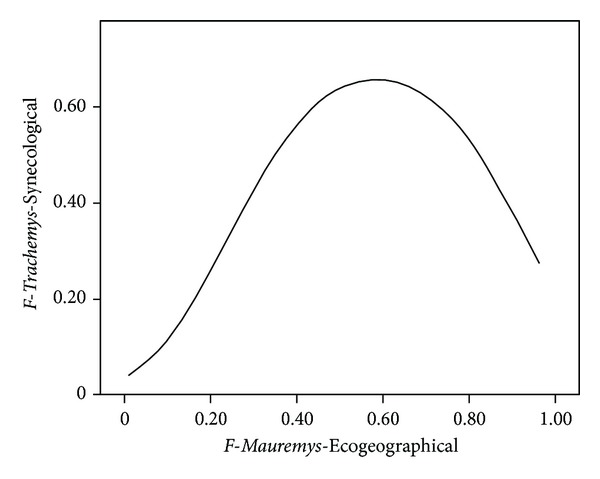
Synecology favourability model (*F*-*Trachemys*-Synecological) for *Trachemys scripta *as a function of ecogeographical favourability model (*F*-*Mauremys*-Ecogeographical) for *Mauremys leprosa*.

**Figure 4:**
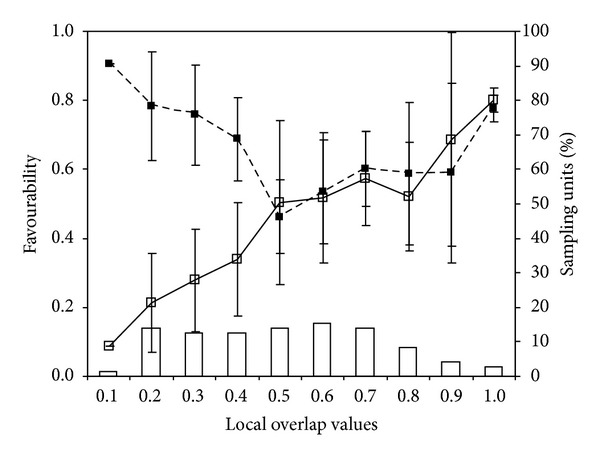
*F*-*Mauremys*-Ecogeographical model (solid lines, filled squares) and *F*-*Trachemys*-Encroachment model (dashed lines, hollow squares). Favourability is shown on the *y*-axis (ranging from 0 to 1); fuzzy overlap between the favourabilities for the two species is shown on the *x*-axis (ranging from 0.1 to 1); columns and sampling unit values represent the percentage of grid cells at each overlap interval.

**Figure 5:**
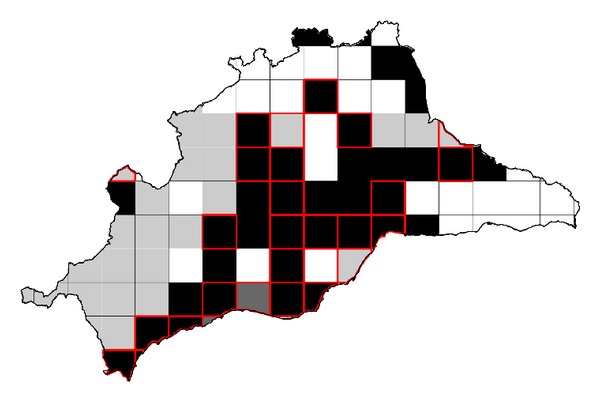
Overlap map. Gray cells indicate areas with overlap values less than 0.4, black cells indicate overlap values between 0.4 and 0.9, and dark gray cells indicate overlap values greater than 0.9. Black cells indicate areas of potential conflict where the red-eared slider could displace the Mediterranean pond turtle. Cells outlined in red show the areas where the red-eared slider is present. Thus, black cells outlined in red indicate current areas of high risk for the Mediterranean pond turtle, and black cells without a red outline indicate areas of high risk for the Mediterranean pond turtle in the future.

The relationship between the *F*-*Trachemys*-Synecology model and the *F*-*Mauremys*-Ecogeographical model is represented in Figure 3. Favourability values for the Mediterranean pond turtle and red-eared slider increased with a positive correlation until reaching a favourability value of 0.6 for both species. Subsequently, although the favourability values for the Mediterranean pond turtle continued to increase, the favourability values for the red-eared slider decreased. In addition, the relationship between the *F*-*Mauremys*-Ecogeographical model and *F*-*Trachemys*-Encroachment model according to the overlap values is represented in Figure 4. In grid cells where overlap values were low (less than 0.4), higher mean favourability values were obtained for the Mediterranean pond turtle than for the red-eared slider. In grid cells with range overlap values between 0.4 and 0.8, the mean favourability values for both species were intermediate and similar. Finally, when overlap values were more than 0.8, the favourability values were very high for both species. The types of relationships between overlap and favourability values for both species are shown in Figure 5, highlighting the areas of conservation concern for the Mediterranean pond turtle due to the encroachment of the red-eared slider.

## 4. Discussion

### 4.1. Distribution Models and the Importance of the Variables Entered for Both Species

The lack of a significant ecogeographical model for the red-eared slider suggests that, not being a native species, its distribution is not yet in equilibrium with the environment which is a common attribute for poor dispersers such as reptiles [31]. However, the significant *F*-*Trachemys*-Encroachment model obtained in our study area proved that the distribution of the red-eared slider is not random, but closely related to the distribution of the Mediterranean pond turtle. Encroachment models of this kind have not been widely used, but we consider that they may become useful tools in studies of native and invasive species with overlapping distributions.

The results of this study revealed differences in environmental requirements between the native and introduced species. Polo-Cavia et al. [12] had already detected physiological differences between both species, with heating and cooling rates being lower for the red-eared slider. In this study, the significant ecogeographical variables for the Mediterranean pond turtle were only associated with natural explanatory factors (climatic, topographical, and geographical). However, the significant variables for the red-eared slider encroachment model also suggested the influence of human activities, implying that the encroachment of the red-eared slider into the range of the Mediterranean pond turtle could be due to the release of pet turtles at water points [6, 7, 32, 33], with natural factors being of secondary importance. Real et al. [33] also found, at national scale, that the distribution pattern of this invasive pond turtle was mediated by human activities. Given that, as Jeschke and Strayer [34] proposed, to be considered invasive, a species should undergo a human-induced introduction in a host area and later should establish and spread, the combination of human and natural factors in the *F*-*Trachemys*-Encroachment model suggest that the human factor points to introduction while the natural factor points to establishment and spread.

The red-eared slider was introduced via pet shops and its release is usually facilitated by their owners due to the nuisance caused when the turtles grow up [33]. Pet species would be difficult to manage if environmental education is lacking. Most people are unaware of the consequences of exotic species introductions and of their own crucial role in this issue. Environmental education is needed in this respect, as an important step in the management of the red-eared slider and other pet species intentionally released.

### 4.2. Interspecific Relationships and Areas Vulnerable to Invasion

Our results indicate that the areas of low favourability for the Mediterranean pond turtle were also areas of low favourability for the red-eared slider (Figure 3). These could be areas whose environmental characteristics make them inappropriate for the community of pond turtles in general. On the other hand, the areas of high favourability for the Mediterranean pond turtle were areas of low favourability for the red-eared slider. In areas where this occurs the presence of the native species could be hindering the settlement of the exotic species. Thus, the exotic species is not expected to generate a significant conservation risk for the native species in these areas. This confirms the finding of Martínez-Silvestre et al. [35], who analysed the interactions between both species, showing that the Mediterranean pond turtle is able to outcompete the red-eared slider and maintain its presence in the habitat, at least in some circumstances.

Richerson and Lum [36] proposed the favourableness hypothesis, suggesting that when environmental conditions approach the lethal limits for a species, organisms must increase the variety of adaptations and energy and material resources devoted to coping with these extreme conditions, to the detriment of those resources devoted to coadaptive accommodation with other species. Conversely, when all environmental conditions approach the optima for the species, organisms can devote a higher amount of energy, matter, and genome to coadaptive adjustments to other species. Consequently, several species may more easily coexist when the environmental conditions are favourable to all of them. Bearing the favourableness hypothesis in mind and making use of the favourability overlap proposed by Acevedo et al. [14], three situations result from the interaction between the two species (see Figure 4). The first situation shows areas where favourability was greater for the Mediterranean pond turtle than for the red-eared slider, corresponding to the areas of low favourability overlap (represented in gray in Figure 5). We expect that the native species will not experience major problems due to the invasive species in these areas. In the remaining areas, the species compete on a similar basis regarding environmental favourability for the two species. However, two different scenarios arise from the favourability overlap shown in Figure 4 (interaction situations 2 and 3). Interaction situation 2 occurs in the areas of intermediate overlap (shown in black in Figure 5), where favourability for both species was intermediate and similar. In these areas, the red-eared slider may outcompete and displace the Mediterranean pond turtle, because the latter is not in optimal environmental conditions. These areas could correspond to areas where the exotic species is regularly released and where favourability for the Mediterranean pond turtle is not very high. Efforts to study, prevent, and mitigate the possible displacement of the native species should be concentrated in these areas, which composed 37.5% of the study area and 51.3% of the area occupied by the Mediterranean pond turtle. Interaction situation 3 affects areas of maximum overlap (just two grids shown in dark grey in Figure 5), where both species have very high favourability and it is more likely that they could coexist according to Richerson and Lum's favourableness hypothesis [36].

Some authors have discussed potential competition between both species [4, 12, 13], but the nature of the competition between them remains poorly understood. Although there is no direct evidence of the red-eared slider and the Mediterranean pond turtle competing for food or natural resources [4, 35], the diet of the red-eared slider strongly overlaps with that of the Mediterranean pond turtle [13]. Polo-Cavia et al. [11, 12, 37] have discussed other types of competition that affect ethological behaviour, showing that, whereas the Mediterranean pond turtle tended to avoid water containing semiochemicals released by the red-eared slider, the red-eared slider was not affected by substances released by the Mediterranean pond turtle. This effect could explain to some extent the displacement of the Mediterranean pond turtle by the red-eared slider. However, none of these competition mechanisms explains the capacity of the Mediterranean pond turtle to hinder, in its most favourable areas, encroachment by invasive pond turtles. The main cause of low favourability for the red-eared slider in most areas favourable to the Mediterranean pond turtle could be the lack of reservoirs. In any case, our methods led to the detection of areas of low concern related to the potential immediate encroachment by the invasive species.

### 4.3. Implications for the Conservation of the Mediterranean Pond Turtle

The approach used in this study can be used to identify areas highly favourable to the red-eared slider, whether or not this species is already present. These areas are of concern for the conservation of the Mediterranean pond turtle, except in the few locations where favourability for the latter species is also high. Thus, this study adds to our knowledge regarding the biogeographical relationship between these native and exotic species and may help to develop strategies to manage the alien species.

Although the nature of competition between these species remains unclear, the Mediterranean pond turtle is at high conservation risk from the red-eared slider. This work not only pinpoints areas where the current presence of the alien species could negatively impact the native species, but also areas where their presence could have a negative impact in the future. The red-eared slider could displace the Mediterranean pond turtle in these areas. Thus, management efforts should be focussed on the areas of highest concern.

The methodology developed in this study could be useful to researchers focused on assessing the potential impact of biological invasions on native species in a global context.

## Figures and Tables

**Table 1:** Variables used to model the distribution of *Trachemys  scripta* and *Mauremys  leprosa*.

Abbreviations	Variable	Abbreviations	Variable
Spatial situation			
La	Latitude (°N)^(1)^	Lo	Longitude (°E)^(1)^

Topography			
*A *	Mean altitude (m)^(2)^	SE	Southward exposure degree^(3)^
*D*_A_	Difference altitude (m) (calculated from altitude)	WE	Westward exposure degree^(3)^
*S *	Slope (°) (calculated from altitude)		

Climate			
*H*_Jul_	Mean relative air humidity in July at 07:00 (%)^(4)^	Dfro	Mean annual number of frost days (minimum temperature ≤ 0°C)^(6)^
*H*_Ran_	Annual relative air humidity range (%) (=|HuJan − HuJul|)	*H*_Jan_	Mean relative air humidity in January at 07:00 (%)^(4)^
*T*_Jan_	Mean temperature in January (°C)^(4)^	RMP	Relative maximum precipitation (=MP24/Prec)
*T*_Jul_	Mean temperature in July (°C)^(4)^	ContI	Continental index^(6)^
Temp	Mean annual temperature (°C)^(4)^	PIrr	Pluviometric irregularity^(7)^
Inso	Mean annual insolation (h/year)^(4)^	ROff	Mean annual runoff (mm)^(5)^
SRad	Mean annual solar radiation (kWh/m^2^/day)^(4)^	DPre	Mean annual number of days with precipitation ≥ 0.1 mm^(4)^
*T*_Ran_	Annual temperature range (°C) (=*T*_Jul_ − *T*_Jan_)	DStrS	Mean annual number of storm in summer^(6)^
MP24	Maximum precipitation in 24 h (mm)^(4)^	Win	Mean annual number of route of winter (km/h)^(6)^
Perm	Soil permeability^(5)^	DOvc	Mean annual number of overcast days^(6)^
HumI	Humidity index^(6)^	DClear	Mean annual number of clear days^(6)^
Prec	Mean annual precipitation (mm)^(4)^	DFog	Mean annual number of fog days^(6)^
AET	Mean annual actual evapotranspiration (mm) (=min⁡[Prec, PET]	DFogW	Mean annual number of fog days in winter^(6)^
PET	Mean annual potential evapotranspiration (mm)^(4)^	DFogS	Mean annual number of fog days in summer^(6)^
DHail	Mean annual number of hail days^(6)^	DStor	Mean annual number of storm days^(6)^
DSno	Mean annual number of snow days^(6)^		

Land cover			
*Natural vegetation *			
PAST	Pasture^(8)^	DForeH	Deciduous forest and hardwood^(8)^
Bush	Bush^(8)^	MxtFor	Mixed forest^(8)^
ScleV	Sclerophyllous vegetation^(8)^	ConiFor	Conifer forest^(8)^
NMead	Natural meadows^(8)^	Mountain	Mountain areas^(8)^
*Crops *			
IHerRice	Irrigated herbaceous crops and rice fields^(8)^	Fruit	Fruit^(8)^
DHerVin	Dry herbaceous crops and vineyard^(8)^	Agricul	Agricultural areas^(8)^
Olive	Olive^(8)^	AgricuLNa	Agricultural land with natural vegetation^(8)^
*Water surfaces *			
LK	Reservoirs and lakes^(8)^	OW	Other wetlands^(8)^
RV	Rivers^(8)^		
*Other use *			
BeachD	Beaches and dunes^(8)^	Mining	Mining area and dumps^(8)^
Urban	Urban zone^(8)^	LinearUr	Urban linear infrastructures^(8)^

Other human activities			
*D*_hi_	Distance to the nearest highway (km)^(1)^	U500	Distance to the nearest urban centre with more than 500 000 inhabitants (km)^(1)^
U100	Distance to the nearest urban centre with more than 100 000 inhabitants (km)^(1)^	HPd	Human population density in 2000 (number of inhabitants/km^2^)^(9)^

(1) Spanish National Geography Institute (IGN), Road map, Iberian Peninsula, Balearics and Canary Island, National Geographic Institute, Ministry of Development, Madrid, Spain, 1999.

(2) United States Geological Survey GTOPO30, Land Processes Distributed Active Archive Center, EROS Data Center, http://lta.cr.usgs.gov/GTOPO30.

(3) T. G. Farr and M. Kobrick, “Shuttle Radar Topography Mission produces a wealth of data,” EOS  Transaction  of  the  American  Geophysical  Union, vol. 81, pp. 583–585, 2000.

(4) I. Font, Climate Atlas of Spain, Madrid, National Meteorology Institute, 1983.

(5) IGME, National Hydrogeological Map (2nd ed), Explanation of useful rainfall maps, of hydrogeological survey and of synthesis of aquifer systems, Madrid, Spanish Mining and Geology Institute, Ministry of Industry and Energy, 1979.

(6) I. Font, Climatology of Spain and Portugal, Ediciones Universidad de Salmanca, 2000.

(7) J. L. Montero de Burgos and J. L. González-Rebollar, Bioclimatic diagrams, Madrid, the National Institute for the Conservation of Nature, España, 1974.

(8) Corine Land Cover CLC2000, Ministry of Development, Government of Spain, http://www.eea.europa.eu/publications/COR0-landcover.

(9) Oak Ridge National Laboratory, LandScan 2000 Global Population Database, Tennesse, USA, Oak Ridge National Laboratory, 2001.

**Table 2:** Comparative assessment of the models developed to *Mauremys leprosa* and *Trachemys  scripta* according to classification, discrimination, and parsimony criteria.

	*F*-*Mauremys*-Ecogeographical	*F*-*Trachemys*-Encroachment	*F*-*Trachemys*-Synecology
Kappa	0.447	0.221	0.167
Sensitivity	0.700	0.667	0.720
Specificity	0.794	0.625	0.519
CCR	0.731	0.635	0.567
AUC	0.806	0.852	0.658
AIC	109.943	66.345	111.963

CCR: correct classification rate; AUC: area under the ROC (receiving operating characteristic); AIC: Akaike information criterion.
